# The Association of Late-Onset Hyponatremia with Feeding Type in Preterm Infants: A Retrospective Study

**DOI:** 10.3390/children13101419

**Published:** 2026-10-16

**Authors:** Supriya Bisht, Lan Jiang, Chenxin Zhang, Mimi Kim, Mariam Susan LaTuga, Mamta Fuloria

**Affiliations:** 1 Division of Newborn Medicine, Department of Pediatrics, University of Mississippi Medical Center, Jackson, MS 39216, USA; 2 Division of Neonatology, Department of Pediatrics, Lurie Children’s Hospital, Northwestern University Feinberg School of Medicine, Chicago, IL 60611, USA; 3 Department of Epidemiology and Population Health, Children’s Hospital at Montefiore, Bronx, NY 10467, USA; 4 Division of Newborn Medicine, Department of Pediatrics, Maria Fareri Children’s Hospital at Westchester Medical Center, New York Medical College, Valhalla, NY 10595, USA; 5 Division of Neonatology, Department of Pediatrics, Children’s Healthcare of Atlanta, Emory University School of Medicine, Atlanta, GA 30322, USA

**Keywords:** preterm neonate, late-onset hyponatremia, expressed breast milk, donor breast milk

## Abstract

**Highlights:**

**What are the main findings?**

- Higher donor breast milk intake was associated with increased odds of late-onset hyponatremia in the primary adjusted analysis, although this association was attenuated after excluding infants receiving prior sodium supplementation.
- Lower gestational age, chlorothiazide use, and mild-to-moderate BPD were additionally independently associated with late-onset hyponatremia.

**What are the implications of the main findings?**

- Preterm infants receiving predominantly donor breast milk, particularly those receiving chlorothiazide, may benefit from close serum sodium monitoring.
- Further studies are needed to identify effective strategies for preventing late-onset hyponatremia in high-risk preterm infants.

**Abstract:**

**Background/Objectives**: Late-onset hyponatremia (LOH) often develops after the second week of life in preterm infants and has been associated with poor growth and adverse neurodevelopmental outcomes. Human milk, including pasteurized donor breast milk (DBM), has less sodium content than preterm infants require. Few studies have examined the association between DBM and LOH. We evaluated the association between feeding type and LOH beyond the second week of life in preterm infants. **Methods**: This was a single-center retrospective cohort study that included preterm neonates born at a gestational age < 32 weeks or birth weight < 1500 g between January 2017 and October 2022. Eligible infants had serum sodium levels measured after 14 days of life and after achieving full enteral feeds. Hyponatremia was defined as serum sodium < 135 mEq/L. **Results**: Of 373 infants, 43% (*n* = 159) had LOH. Multivariable regression analysis suggested that each 10% increase in DBM intake was associated with higher odds of LOH (adjusted OR 1.08, 95% CI 1.01–1.16; *p* = 0.022). Lower gestational age, chlorothiazide use, and mild-to-moderate bronchopulmonary dysplasia (BPD) were also associated with higher odds of LOH. The use of furosemide, formula, and hemodynamically significant patent ductus arteriosus were not associated with LOH. **Conclusions**: Higher DBM intake, lower gestational age, chlorothiazide use, and mild-to-moderate BPD were associated with LOH in the primary multivariable analysis. However, the DBM association was attenuated and not statistically significant after excluding infants who had received prior sodium supplementation. Preterm infants receiving predominantly DBM, particularly those treated with chlorothiazide, may benefit from close serum sodium monitoring.

## 1. Introduction

Sodium is a critical element for neonatal nutrition, development, and metabolism. It maintains extracellular fluid volume [1,2], promotes protein synthesis, and plays a vital role in the development of the central nervous system. Furthermore, sodium is a key constituent of bone, cartilage, and connective tissues [3,4].

Hyponatremia is defined as a serum sodium level < 135 mEq/L [5,6]. The overall incidence of hyponatremia in preterm infants admitted to the Neonatal Intensive Care Unit (NICU) is approximately 20–30% [5,7]. The incidence of hyponatremia among very-low-birth-weight (VLBW) infants is reported to be as high as 62.5% [8].

Late-onset hyponatremia (LOH), by definition, develops after the second week of life and is associated with poor growth and adverse neurodevelopmental outcomes in preterm infants [9]. It results from decreased sodium intake, impaired gastrointestinal sodium absorption, and immature water and sodium homeostasis in the kidneys [3,5,6,8]. The duration and severity of LOH correlate with prematurity, morbidities such as bronchopulmonary dysplasia (BPD), and renal immaturity. Medications such as caffeine, diuretics, and enteral feeding types have also been linked with LOH [3,10].

Ideally, VLBW infants should be exclusively fed breast milk. The sodium content of the mother’s own unfortified expressed breast milk (EBM), at a volume of 180 mL/kg/day, provides approximately 1–2 mEq/kg/day of sodium [7,11], which is significantly lower than the required 3–7 mEq/kg/day [10]. The American Academy of Pediatrics recommends fortifying maternal milk for VLBW infants to provide appropriate nutrient intake [12]. Even with fortification, EBM does not meet the sodium requirement of preterm infants [7,8,11]. If EBM is unavailable, VLBW infants should be fed fortified pasteurized donor breast milk (DBM) [12]. DBM is primarily obtained from mothers of term infants and has a composition similar as mature milk [13]. Compared with preterm milk, DBM is reported to be significantly lower in nutrients such as protein, sodium, chloride, potassium, and zinc [14]. Storage, contact with containers, and heat processing can further affect the components and nutritional content of DBM [13,15]. Even with fortification, the sodium content of DBM remains below the recommended range for preterm infants [16,17]. Preterm milk formulas also have varying sodium content and fall below the physiological requirements of preterm infants [16].

While previous studies have explored the role of breast milk and formula in sodium homeostasis in preterm infants, few studies have examined the association between DBM and LOH. This study aims to address this gap by evaluating the relationship between feeding type and LOH in preterm infants after 14 days of life. We also analyzed clinical characteristics, growth outcomes, and other factors commonly associated with LOH, like respiratory distress syndrome (RDS), diuretic use, hemodynamically significant patent ductus arteriosus (hs-PDA), culture-positive sepsis, and BPD.

## 2. Materials and Methods

### 2.1. Study Population

This was a retrospective single-center cohort study that included preterm neonates born at gestational age (GA) < 32 weeks or BW < 1500 g, between January 2017 and October 2022 at Montefiore Medical Center—Jack D. Weiler NICU. Albert Einstein College of Medicine-Montefiore Medical Center Institutional Review Board reviewed the study and determined that it met the criteria for exemption.

All infants meeting the eligibility criteria during the study period were included. Infants with genetic syndromes, congenital gastrointestinal anomalies, major gastrointestinal surgery, surgical necrotizing enterocolitis (NEC), and major congenital renal anomalies were excluded from the study. Infants receiving enteral sodium supplementation with serum sodium ≥ 135 mEq/L were also excluded, as their underlying sodium status could not be reliably determined. Infants receiving sodium supplementation who remained hyponatremic (serum sodium < 135 mEq/L) were retained and classified as LOH.

### 2.2. Data Collection

The NICU encouraged EBM feeding. If EBM was unavailable, DBM was provided after parents consented to DBM usage. Breast milk was fortified to 24 kcal/oz once enteral nutrition reached 80 mL/kg/day. Parenteral nutrition or intravenous fluids were discontinued once enteral feed volume reached 120 mL/kg/day. The average enteral feeding goal was 160 mL/kg/day. The NICU used Similac^®^ human milk fortifier hydrolyzed protein concentrated liquid for fortification. Fortification was adjusted to 26 kcal/oz for infants with suboptimal growth, as per standard unit practice. Infants were included who had a serum sodium level after 14 days of life and were on full enteral feeds. Full enteral feeds were defined as enteral intake of ≥140 mL/kg/day of EBM, DBM, or preterm formula for at least 5 days, with no parenteral nutrition or intravenous fluids containing sodium.

Only the first eligible serum sodium value was used for classification -defined as the first measurement obtained after 14 days of life, after achieving full enteral feeds as defined above, and before 36 weeks postmenstrual age (PMA). The 36-week PMA cutoff served as the upper boundary of the measurement window.

BPD is classified at 36 weeks PMA as: Grade 1 or Mild BPD if infant requires ≤2 L/min nasal cannula, Grade 2 or Moderate if >2 L/min nasal cannula or other non-invasive ventilation support, and Grade 3 or severe BPD if receiving invasive mechanical ventilation [18]. The minimum discharge age in the NICU was 35 weeks PMA. Infants discharged before 36 weeks PMA were on room air and were classified as having no BPD; no infants with Grade II or higher BPD were discharged before 36 weeks PMA.

Clinical characteristics of the study patients were obtained from an electronic health records system. Feeding details five days prior to the documented first serum sodium value were obtained, including type of feed, feeding volume, caloric intake, fortification type, and use of liquid protein (0.5–1 g/kg/day) and medium-chain triglyceride (MCT, as 0.5 mL per 30 mL of milk). Additionally, respiratory support details, serum creatinine, sodium supplementation, and packed red blood cell (PRBC) transfusion were also documented.

### 2.3. Outcomes

The primary outcome of the study was to evaluate the association of LOH in preterm infants with GA < 32 weeks or BW < 1500 g, on full enteral feeds with the following types of feeds—EBM, DBM, or preterm formula.

The secondary outcome was to compare clinical characteristics, growth outcomes, and morbidities associated with prematurity along with their management, including RDS, diuretic use (short-acting such as furosemide and long-acting such as chlorothiazide), hs-PDA, culture-positive sepsis, and BPD.

### 2.4. Statistical Analysis

The primary predictor variables were milk intake: EBM, DBM, and formula, presented as mL/kg/day or as a percentage of total milk intake. The primary outcome measured in the study was the presence of LOH.

Continuous data were summarized using standard descriptive statistics, including means, standard deviations, medians, interquartile ranges, and the discrete variables as absolute and relative frequencies. To compare continuous variables, the Wilcoxon rank-sum test was used; discrete variables were compared using the Pearson Chi-square test or Fisher’s exact test, as appropriate.

Univariate and multivariable logistic regression were performed to estimate odds ratios and their corresponding confidence intervals (CI). Covariates for the multivariable analysis were selected based on their clinical relevance, prior literature, and univariable analysis. The model was adjusted for GA, race, furosemide, chlorothiazide, BPD, and hs-PDA. BW was not included because of its strong correlation with GA and potential collinearity. All statistical tests were two-sided, and significance was indicated by *p*  <  0.05. The statistical analysis was performed using R language version 4.3.1 (R Core Team, 2023).

The initial sample size estimate was based on approximately 400 eligible infants and was expected to provide at least 80% power (two-sided α = 0.05) to detect a 14.5-percentage-point difference in LOH between the planned EBM and non-EBM groups. As most infants received a combination of EBM, DBM, and formula, the final analysis used the percentage of DBM intake as a continuous exposure to better reflect actual feeding practices and utilize the available feeding data. The original power calculation therefore does not directly apply to the final analysis using DBM intake as a continuous exposure, and a separate power calculation for this analysis was not performed. Data were complete for all analyzed variables.

## 3. Results

**Figure 1:**
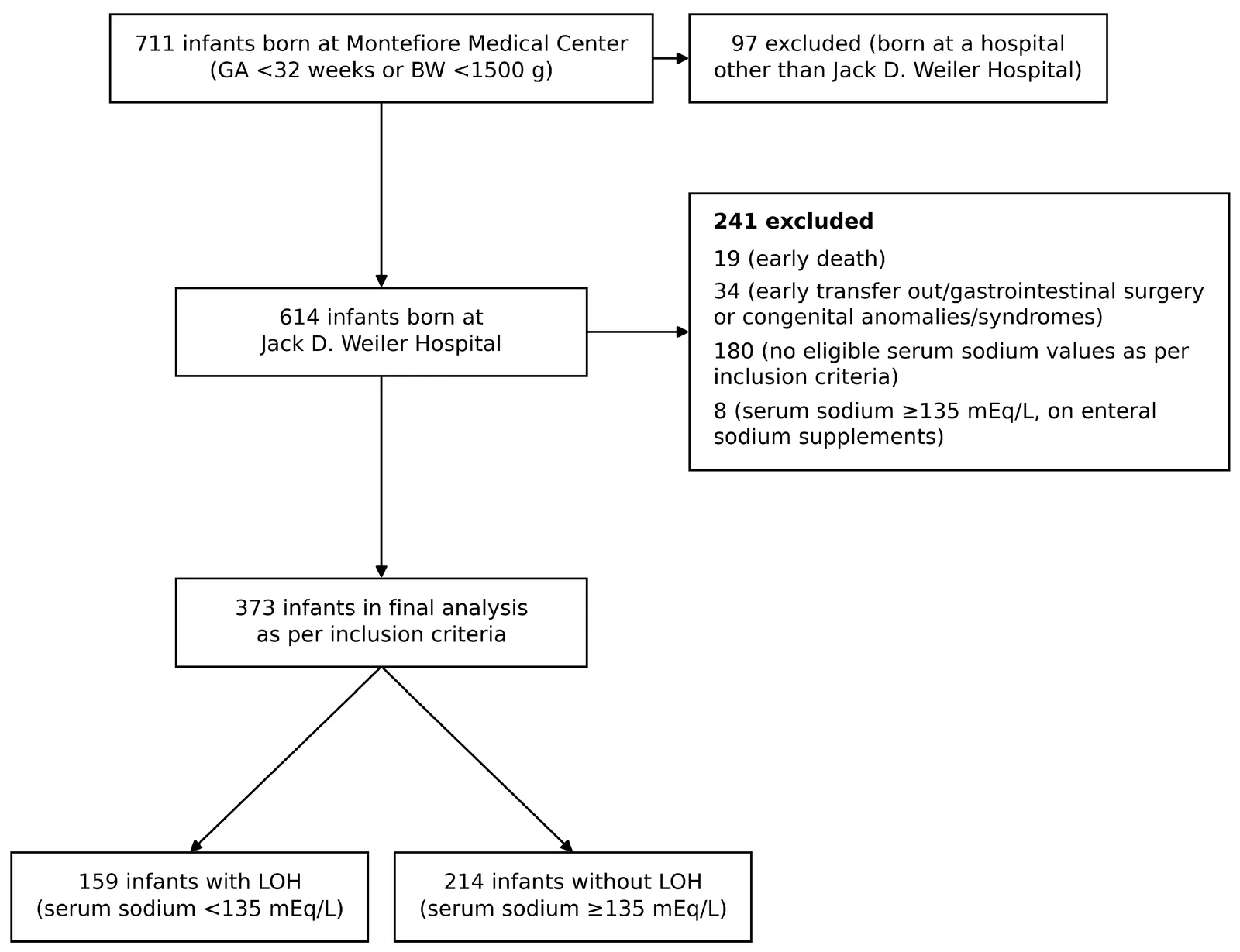
Flowchart of infants in the study.

During the study period, a total of 711 preterm infants were admitted at the Montefiore Medical Center (Figure 1). Overall, 373 infants were included in the final analysis, of which 43% (*n* = 159) had LOH.

### 3.1. Risk Factors for Hyponatremia

Infants with LOH were born at lower GA and had lower BW than infants without LOH (Table 1).

Serum sodium was measured at a lower mean PMA for infants with LOH than for infants without LOH (Table 2). The average enteral intake was comparable between the two groups. The majority of the infants (*n* = 304, 82%) received 24 kcal/ounce feeds, while 15% (*n* = 56) received 26 kcal/ounce feeds. Among infants with LOH, 31% (*n* = 50) received prior sodium supplements, which increased to 69% (*n* = 110) after serum sodium levels were obtained (Table 2).

As shown in Table 2, infants with LOH received a significantly higher percentage of DBM and a lower percentage of formula compared to those without LOH. Infants with LOH had higher rates of RDS requiring surfactant, hs-PDA, BPD, culture-positive sepsis, increased serum creatinine, increased number of PRBC transfusions, increased use of diuretics such as furosemide and chlorothiazide, higher oxygen requirement, level of respiratory support, and prolonged NICU stay (Table 3).

**Figure 2:**
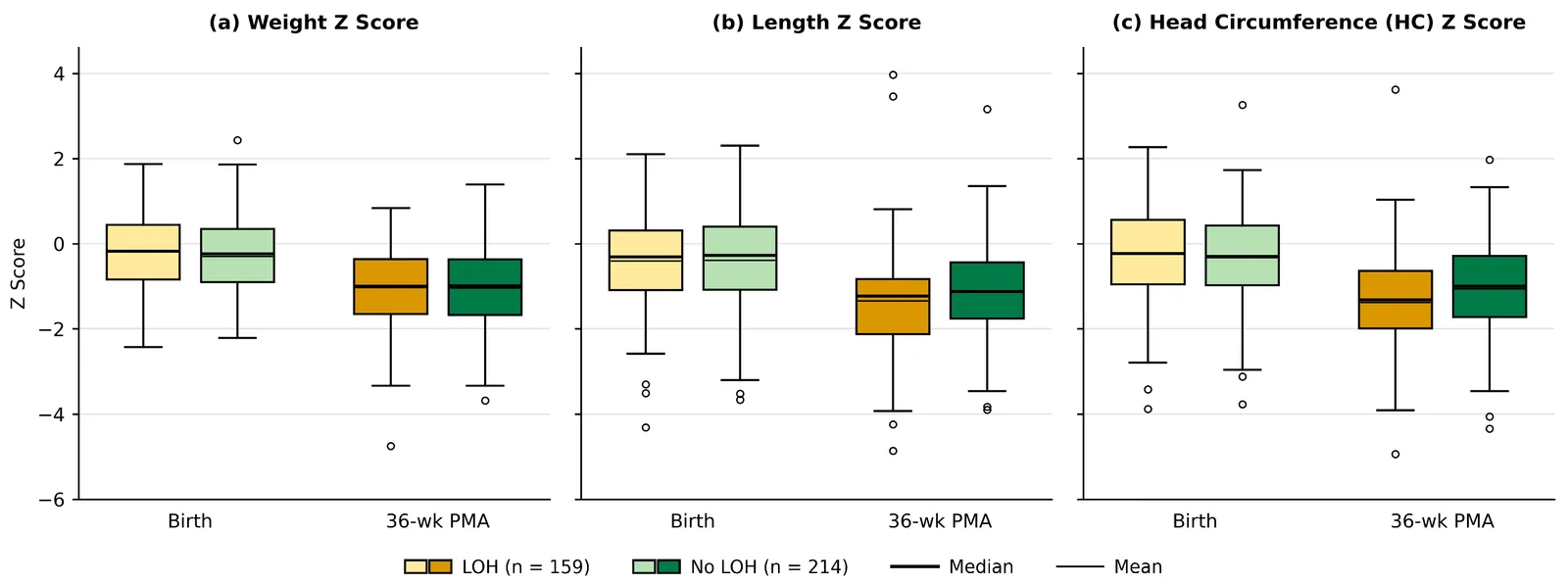
Weight, length and head circumference (HC) Z-scores at birth and at 36 weeks postmenstrual age (PMA) in preterm infants with and without late-onset hyponatremia (LOH).

Infants with LOH had significantly lower Z scores (Fenton 2013 growth charts used) for both length and head circumference (HC) at 36 weeks PMA when compared to infants without LOH. Furthermore, the Z scores for length and head circumference in the LOH group decreased significantly from birth to 36 weeks PMA, compared with infants without LOH (Figure 2). Additional data is described in Table S1.

### 3.2. Univariable Regression Analysis

Univariable regression analysis showed that lower birth GA and BW, lower PMA at the time of serum sodium measurement, lower daily weight, and less formula use were associated with LOH. A higher percentage of DBM use, increased use of furosemide and chlorothiazide, and presence of comorbidities such as BPD and hs-PDA were also associated with LOH (Table 4).

### 3.3. Multivariable Regression Analysis

Multivariable regression analysis showed that each 10% increase in DBM intake was associated with a 1.08 times higher odds ratio of developing LOH. In addition, LOH was independently associated with lower GA, use of chlorothiazide, mild BPD, and moderate BPD. The use of furosemide, formula, and hs-PDA were not associated with LOH (Table 5) in the multivariable regression analysis.

### 3.4. Sensitivity and Subgroup Analysis

In the sensitivity analysis (Table S2) excluding infants receiving either diuretic (n = 283), the association remained statistically significant (OR 1.13 per 10% increase in DBM, 95% CI 1.05–1.23; *p* = 0.002). In a separate sensitivity analysis excluding the 50 infants in the LOH group receiving enteral sodium supplementation before serum sodium measurement (n = 323), the association between DBM intake and LOH was attenuated and no longer statistically significant (OR 1.05 per 10% increase in DBM, 95% CI 0.98–1.14; *p* = 0.166). In the exploratory subgroup analyses stratified by gestational age, the association between DBM intake and LOH remained positive but did not reach statistical significance in infants ≤28 weeks (OR 1.08 per 10% increase, 95% CI 0.98–1.19; *p* = 0.127) or in infants >28 weeks (OR 1.11, 95% CI 0.99–1.25; *p* = 0.077) (Table S2).

The predicted probability of LOH increased from 16.9% (95% CI 6.1–39.0%) at 0% DBM intake to 31.4% (95% CI 11.3–62.3%) at 100% DBM intake, holding all other covariates at reference values (Figure S1).

## 4. Discussion

This study is among the few to evaluate the association between LOH and different feeding types in preterm infants (breast milk vs. formula), further categorizing breast milk as either EBM or DBM. Our findings suggest a dose-dependent relationship between DBM intake and LOH in preterm infants with GA < 32 weeks or BW < 1500 g in the primary analysis, making this a novel contribution to the existing literature on the relationship between feeding type and LOH in preterm infants.

Consistent with prior studies reporting a prevalence rate of LOH ranging between 33–70%, 43% of preterm infants in our study population developed LOH [10]. LOH is an important concern in preterm infants due to its association with hearing loss, adverse neurodevelopmental outcomes, and slow weight gain [1,6]. Hence, it is important to maintain serum sodium levels within the normal range in preterm infants.

We observed significantly lower mean birth GA and BW in the LOH group as compared to the no-LOH group. Previous studies have similarly shown that LOH is associated with a lower GA and BW [3,7,8]. Lower GA is associated with LOH [5], likely because of an immature renal system and higher urine sodium loss [19].

In our study population, the mean serum sodium level in the LOH group was 132.6 (±1.6) mEq/L compared to 138.3 (±2.2) mEq/L in the no-LOH group. The serum sodium level was measured at 33.5 (±11.8) days of life in the LOH group vs. 28.4 (±11.3) days of life in the no-LOH group. In one study, at the time of LOH diagnosis, the mean serum sodium level was 129.3 ±3.4 mEq/L and the mean age was 29.6 ±2.2 days [5].

Similar to previous studies, the infants in the LOH group were more likely to be born to mothers with chorioamnionitis (13%). They also had a lower 5-min Apgar score compared to those with no-LOH group. Maternal premature rupture of the membranes and use of prenatal antibiotics have previously been reported to be more common in infants with LOH [3,7]. Maternal hypertension was noted in 36% of the LOH group.

The day of life of starting feeds (including colostrum care) was delayed in the LOH group (Day 2 compared to 1.6 days in the no-LOH group). This finding is likely related to the lower GA and BW in the LOH group since the degree of prematurity often directly correlates with clinical instability. A study showed similar delay in starting enteral feeds, with an average of 6.4 days in the hyponatremia group compared to 5.8 days in the no-LOH group [8].

Infants with LOH in our study had greater illness severity. A higher proportion of the infants with LOH required surfactant administration for RDS in our study and were more likely to be diagnosed with hs-PDA, BPD, and culture-positive sepsis. They also had higher serum creatinine levels, needed PRBC transfusion, were treated with diuretics (furosemide, chlorothiazide), had a higher oxygen requirement, needed more respiratory support, and had longer NICU hospitalization. These findings are similar to other studies, in which factors such as RDS, PDA requiring treatment, postnatal sepsis, and the use of furosemide were significantly more common in the LOH group [3,7]. Another study observed that PDA ligation, sepsis, use of furosemide, and BPD occurred more commonly in the severe hyponatremia group (serum sodium < 130 mEq/L) compared to mild LOH [5], indicating that higher illness in preterm infants is linked with more severe LOH.

As a descriptive secondary finding, infants with LOH had a lower birth GA and BW. At 36 weeks PMA, the LOH group in our study had significantly lower Z scores for length and HC, as well as a significant decrease in Z score change (both length and HC) from birth to 36 weeks PMA, compared to the no-LOH group (Figure 2). Postnatal growth is influenced by multiple nutritional factors, including calorie intake as well as macro-and micronutrient intake [20]. Since growth was a secondary outcome for our study, we did not assess protein intake in our study patients, and regression analysis was not performed to determine the independent association between LOH and growth parameters at 36 weeks PMA. These findings should be interpreted as descriptive only. Previous studies have reported inconsistent findings in this regard. In some studies, growth parameters are negatively affected when infants receive a majority of DBM feeds [21,22]. Other studies suggested that VLBW infants receiving either EBM or DBM exhibited similar growth velocities [23], and DBM did not significantly affect the average change in the anthropometric parameters in these infants [24]. The impact of feeding type on growth could be attributed to differences in NICU feeding policies and to variability in DBM’s nutritional and caloric status.

In our study, multivariable regression analysis showed that increased DBM intake, lower GA, chlorothiazide use, and mild-to-moderate BPD were associated with higher odds of LOH. The use of furosemide, formula feeds, and hs-PDA was not associated with LOH in the adjusted analysis (furosemide was used only for short-term treatment). Sensitivity analyses showed that the association between DBM intake and LOH remained statistically significant after excluding infants receiving diuretics, suggesting that the observed association was not explained solely by diuretic use. However, after excluding infants receiving enteral sodium supplementation, the association was no longer statistically significant. These infants had persistent hyponatremia despite receiving enteral sodium supplementation, suggesting ongoing sodium deficits or increased sodium requirements. Excluding these infants removed an important subgroup from the analysis. Together, these findings suggest that the association between DBM intake and LOH may be influenced by the clinical characteristics and sodium supplementation practices of the study population. These findings should be interpreted with caution given the retrospective study design and the possibility of confounding. In subgroup analyses by gestational age (GA ≤ 28 weeks and >28 weeks), the association remained in the same direction but did not reach statistical significance in either group. The predicted probability of LOH increased from 16.9% with no DBM intake to 31.4% with 100% DBM intake, further demonstrating the clinical importance of the observed association.

While previous studies have identified risk factors for LOH and evaluated the role of breast milk and formula in the development of LOH, the majority of these studies did not focus on specific breast milk types (i.e., EBM vs. DBM). A recent smaller study found that preterm infants with hyponatremia had received more maternal breast milk compared to DBM, which differs from our findings. This discrepancy may be related to differences in gestational age, study design, and how donor milk exposure was measured, as well as possible variations in milk fortification practices and sodium content [25]. Breastmilk feeding, even with fortification (compared to formula), has been associated with hyponatremia [3,6,7,8]. Our results demonstrate that higher DBM intake is associated with LOH in preterm infants. The dose-dependent nature of this association is important. This finding is likely due to the lower sodium content and potential nutritional variability of DBM. However, the sodium content of the DBM used in our study was not measured.

As LOH is associated with adverse outcomes among preterm infants, in infants who are receiving a higher proportion of DBM feeds and treated with diuretics such as chlorothiazide, serum sodium levels should be monitored closely. Recent retrospective studies have suggested that enteral sodium supplementation improves growth parameters in preterm infants [26,27] with reduced time on invasive mechanical ventilation without increased incidence of hypernatremia, hypertension, BPD or NEC [27]. In a randomized controlled study, early sodium supplementation was associated with improved weight gain and linear growth in very preterm infants [28]. This further emphasizes the importance of avoiding hyponatremia in high-risk preterm infants and its associated complications. Current NICU practices regarding routine sodium surveillance and supplementation vary among institutions. Some centers routinely monitor serum sodium or urine sodium concentrations in preterm infants and provide enteral sodium supplementation when deficits are identified. The clinical focus should be on ensuring adequate sodium intake and timely electrolyte monitoring in preterm infants receiving a higher proportion of DBM, rather than on the feeding type itself.

The major strength of this study is the separation of human milk into EBM and DBM, quantified both volumetrically and as a proportion of total intake, in a reasonably large single-center cohort with detailed feeding and respiratory data.

Our study has several limitations, including its retrospective cohort design. Approximately one-third of the infants were excluded because no serum sodium level was available after two weeks of life. These infants may have been healthier or more mature, potentially resulting in a study population with a higher baseline risk of LOH. However, demographic data for the excluded infants were unavailable to confirm this possibility. Thirty-one percent of infants in the LOH group were already receiving enteral sodium supplements based on sodium values in point-of-care expanded blood gas testing. Our study excluded infants on sodium supplementation with normal serum sodium levels, while those with LOH were included. This may have caused selection bias, affecting the observed prevalence of LOH and its association with DBM intake. Feeding data were captured only over the five days preceding the sodium measurement, which may not fully reflect cumulative sodium intake. The use of a single time-point sodium measurement may underestimate the true prevalence of LOH. Although the multivariable analysis adjusted for several clinically relevant factors, residual confounding remains possible. Total sodium intake from milk, fortification and supplementation, caffeine exposure, fluid balance/urine output, and duration of prior parenteral nutrition were not included in the analysis and could contribute to the observed association between DBM intake and LOH.

This is a single-center study, and as such, the results may not be universally applicable, particularly in settings with different feeding protocols and institutions caring for infants with lower illness severity. Despite these limitations, periodic surveillance of serum sodium levels for infants receiving mainly DBM feeds and ongoing diuretics is primarily warranted.

## 5. Conclusions

In this single-center retrospective cohort of preterm infants born at GA < 32 weeks or BW < 1500 g, higher DBM intake, lower birth GA, chlorothiazide use, and mild-to-moderate BPD were associated with LOH in the primary multivariable analysis. However, the association between DBM intake and LOH was attenuated and was no longer statistically significant after excluding infants receiving prior sodium supplementation, suggesting that the findings should be interpreted with caution. These findings highlight the importance of monitoring serum sodium levels in preterm infants receiving predominantly DBM, particularly those with additional risk factors. Further prospective studies assessing sodium intake from different feeding types, sodium supplementation practices, and serial serum sodium levels are needed to better understand this association.

## Figures and Tables

**Table 1:** Demographic Characteristics.

Variables	Total *N* = 373	No LOH *N* = 214 ^1^	LOH *N* = 159 ^1^	*p*-Value ^2^
GA at birth Mean (±SD)	28.3 (±2.5)	29.4 (±2.1)	26.8 (±2.2)	<0.001
Race				
White	34 (9.1%)	21 (9.8%)	13 (8.2%)	0.062
Black	138 (37%)	68 (32%)	70 (44%)	
Asian	13 (3.5%)	6 (2.8%)	7 (4.4%)	
Other	188 (50%)	119 (56%)	69 (43%)	
Ethnicity				
Non-Hispanic	199 (53%)	107 (50%)	92 (58%)	0.12
Hispanic	174(47%)	107 (50%)	67 (42%)	
Gender: Male	195 (52%)	106 (50%)	89 (56%)	0.22
BW (g) Mean (±SD)	1058.2 (±341.3)	1185.6 (±325.6)	886.6 (±282.4)	<0.001
BW percentile Mean(±SD)	42.3 (±26.8)	41.1 (±25.8)	43.8 (±28.0)	0.41
Cesarian delivery	258 (69%)	150 (70%)	108 (68%)	0.65
Chorioamnionitis	35 (9.4%)	14 (6.5%)	21 (13%)	0.028
Maternal PPROM *	111 (30%)	63 (30%)	48 (31%)	0.77
Maternal antibiotics received	193 (52%)	105 (49%)	88 (55%)	0.23
Maternal gestational diabetes	58 (16%)	37 (17%)	21 (13%)	0.27
Maternal hypertension	158 (43%)	101 (47%)	57 (36%)	0.029
Antenatal corticosteroids	286 (77%)	162 (76%)	124 (78%)	0.88
Apgar 5 min Mean (±SD)	7.7 (±1.3)	8.1 (±1.1)	7.3 (±1.3)	<0.001
Day of starting feeds Mean (±SD)	1.8 (±1.2)	1.6 (±1.0)	2.0 (±1.4)	0.029
Type of feeds started				
EBM	297 (80%)	165 (77%)	132 (83%)	0.18
DBM	65 (17%)	40 (19%)	25 (16%)	
Formula	11 (2.9%)	9 (4.2%)	2 (1.3%)	

^1^ Values are mean (SD), or count (%). ^2^ Wilcoxon rank sum test; Pearson’s Chi-squared test; Fisher’s exact test. * PPROM: Preterm prelabor rupture of membranes.

**Table 2:** Nutritional characteristics of infants at the time of serum sodium measurement.

Variables	Total *N* = 373 ^1^	No LOH *N* = 214 ^1^	LOH *N* = 159 ^1^	*p*-Value ^2^
PMA (weeks), Mean (±SD)	32.6 (±2.0)	33.5 (±1.5)	31.5 (±2.0)	<0.001
Day of life at serum sodium measurement Mean (±SD)	30.5 (±11.8)	28.4(±11.3)	33.5 (±11.8)	<0.001
Serum sodium (mEq/L) Mean (±SD)	135.9 (±3.5)	138.3 (±2.2)	132.6 (±1.6)	<0.001
On prior enteral sodium supplement	50 (13%)	0 (0%)	50 (31%)	<0.001
Enteral sodium started afterwards	110 (29%)	0 (0%)	110 (69%)	<0.001
Daily weight (kg) Mean (Range)	1.47 (0.63, 2.94)	1.60 (0.79, 2.94)	1.31 (0.63, 2.32)	<0.001
Milk Fortification				
24 kcal/oz	304 (82%)	178 (83%)	126 (79%)	0.020
22 kcal/oz	13 (3.5%)	11 (5.1%)	2 (1.3%)	
26 kcal/oz	56 (15%)	25 (12%)	31 (19%)	
Liquid Protein	82 (22%)	49 (23%)	33 (21%)	0.62
MCT Oil	14 (3.8%)	8 (3.7%)	6 (3.8%)	0.99
EBM (mL/kg/day) Mean (Range)	101.8 (0.0, 171.0)	106.1 (0.0, 171.0)	95.9 (0.0, 168.2)	0.11
DBM (mL/kg/day) Mean (Range)	34.4 (0.0, 164.8)	24.65 (0.0, 159.6)	47.58 (0.0, 164.8)	0.004
Formula (mL/kg/day) Mean (Range)	18.8(0.0, 198.3)	25.4 (0.0, 198.3)	9.9(0.0, 160.0)	<0.001
EBM (%) Mean (Range)	65.8 (0–100%)	68.2 (0–100%)	62.6 (0–100%)	0.45
DBM (%) Mean (Range)	22.2 (0–100%)	15.9 (0–100%)	30.8 (0–100%)	0.004
Formula (%) Mean (Range)	12.0 (0–100%)	15.9 (0–100%)	6.6 (0–100%)	<0.001

^1^ Values are mean (±SD or range), or count (%). ^2^ Wilcoxon rank sum test; Pearson’s Chi-squared test; Fisher’s exact test.

**Table 3:** Clinical characteristics of infants.

Variables	Total *N* = 373 ^1^	No LOH *N* = 214 ^1^	LOH *N* = 159 ^1^	*p*-Value ^2^
RDS requiring surfactant	194 (52%)	80 (37%)	114 (72%)	<0.001
hs-PDA	105 (28%)	32 (15%)	73 (46%)	<0.001
PDA treatment				
Medical	75 (20%)	20 (9.3%)	55 (35%)	<0.001
Transcatheter/surgical closure	19 (5.1%)	5 (2.3%)	14 (8.8%)	
No treatment	279 (75%)	189 (88%)	90 (57%)	
Medical NEC	18 (4.8%)	8 (3.7%)	10 (6.3%)	0.26
BPD				
No BPD	101 (27%)	92 (43%)	9 (5.7%)	<0.001
Mild	142 (38%)	78 (36%)	64 (40%)	
Moderate	119 (32%)	41 (19%)	78 (49%)	
Severe	11 (2.9%)	3 (1.4%)	8 (5.0%)	
Serum creatinine (mg/dL) Mean (±SD)	0.46 (±0.12)	0.44 (±0.10)	0.49 (±0.13)	0.001
PRBC transfusion	35 (9.4%)	6 (2.8%)	29 (18%)	<0.001
Respiratory support *				
HFNC/Nasal Cannula	37 (9.9%)	25 (12%)	12 (7.5%)	<0.001
CPAP	184 (49%)	101 (47%)	83 (52%)	
NIPPV	40 (11%)	11 (5.1%)	29 (18%)	
Invasive Ventilation	33 (8.8%)	5 (2.3%)	28 (18%)	
Room Air	79 (21%)	72 (34%)	7 (4.4%)	
FiO_2_: Mean (Range)	23.2 (21.0, 50.0)	21.4 (21.0, 35.0)	25.6 (21.0, 50.0)	<0.001
Furosemide received	29 (7.8%)	9 (4.2%)	20 (13%)	0.003
Chlorothiazide received	74 (20%)	15 (7.0%)	59 (37%)	<0.001
Sepsis (Culture-positive)	4 (1.1%)	0 (0%)	4 (2.5%)	0.032
NICU Stay (days) Mean (Range)	77.9 (20.0, 242.0)	63.4 (20.0, 147.0)	97.3 (23.0, 242.0)	<0.001
Infant Disposition				
Home	303 (81%)	194 (91%)	109 (69%)	<0.001
Transfer to rehabilitation	61 (16%)	16 (7.5%)	45 (28%)	
Transfer to another hospital	9 (2.4%)	4 (1.9%)	5 (3.1%)	

^1^ Values are mean (±SD or range), or count (%). ^2^ Wilcoxon rank sum test; Pearson’s Chi-squared test; Fisher’s exact test. * HFNC (high-flow nasal cannula); CPAP (continuous positive airway pressure); NIPPV (nasal intermittent positive pressure ventilation).

**Table 4:** Univariable Logistic Regression of Late-Onset Hyponatremia.

Characteristic	OR ^1^	95% CI ^1^	*p*-Value
GA at birth	0.5847	0.5175, 0.6544	<0.0001
Race			
White	reference	—	
Black	1.6629	0.7794, 3.6601	0.1943
Asian	1.8846	0.5174, 7.0941	0.3361
Other	0.9367	0.4457, 2.0308	0.8647
Gender: Female	0.7720	0.5104, 1.1648	0.2183
BW (g)	0.9967	0.9959, 0.9975	<0.0001
BW (per 100 g increase)	0.7200	0.6600, 0.7804	<0.0001
PMA at serum sodium	0.5321	0.4558, 0.6135	<0.0001
Intake	0.9584	0.9317, 0.9836	0.0021
Daily Weight	0.0968	0.0479, 0.1866	<0.0001
Milk Fortification			
24 kcal/oz	reference	—	
22 kcal/oz	0.2569	0.0393, 0.9777	0.0804
26 kcal/oz	1.7517	0.9886, 3.1318	0.0557
Liquid Protein used	0.8819	0.5324, 1.4470	0.6213
MCT Oil Used	1.0098	0.3265, 2.9628	0.9859
EBM (per mL/kg/day)	0.9977	0.9946, 1.0008	0.1417
EBM (per 1%)	0.9970	0.9923, 1.0018	0.2168
EBM (per 10%)	0.9705	0.9254, 1.0178	0.2168
DBM (per mL/kg/day)	1.0067	1.0031, 1.0103	0.0003
DBM (per 1%)	1.0104	1.0049, 1.0161	0.0002
DBM (per 10%)	1.1093	1.0504, 1.1732	0.0002
Formula (per mL/kg/day)	0.9916	0.9859, 0.9967	0.0022
Formula (per 1%)	0.9878	0.9791, 0.9956	0.0035
Formula (per 10%)	0.8843	0.8097, 0.9564	0.0035
BPD			
Mild	8.3875	4.1012, 19.0558	<0.0001
Moderate	19.4472	9.3025, 45.0904	<0.0001
Severe	27.2593	6.6664, 142.9932	<0.0001
Sepsis (culture-positive) *	NE	0.0000 NA	0.9826
hs-PDA present	4.8278	2.9870, 7.9490	<0.0001
Furosemide received	3.2774	1.4900, 7.7658	0.0043
Chlorothiazide received	7.8273	4.3324, 14.9490	<0.0001

^1^ OR = Odds Ratio; CI = Confidence Interval. * Not estimable due to zero event occurring in the no-LOH group.

**Table 5:** Multivariable Logistic Regression of Late-Onset Hyponatremia.

Characteristic	OR ^1^	95% CI ^1^	*p*-Value
DBM (per 1%)	1.0082	1.0013, 1.0153	0.0217
DBM (per 10%)	1.0848	1.0127, 1.1640	0.0217
Formula (per 1%)	0.9940	0.9834, 1.0043	0.2594
Formula (per 10%)	0.9418	0.8463, 1.0437	0.2594
GA at birth	0.7348	0.6294, 0.8521	<0.0001
Race			
White	reference	—	
Black	0.9894	0.3656, 2.7370	0.9834
Asian	2.1799	0.4250, 11.6660	0.3531
Other	0.6059	0.2277, 1.6499	0.3191
Furosemide received	1.3784	0.5264, 3.8462	0.5231
Chlorothiazide received	3.2817	1.6074, 6.9978	0.0015
BPD			
Mild	2.9077	1.2639, 7.2336	0.0155
Moderate	3.8238	1.4718, 10.5046	0.0070
Severe	2.1640	0.3938, 13.8656	0.3863
hs-PDA	1.2048	0.6326, 2.2769	0.5672

^1^ OR = Odds Ratio; CI = Confidence Interval.

## Data Availability

The data presented in this study are available from the corresponding author upon reasonable request and with permission from the Institutional Review Board, due to patient confidentiality and privacy restrictions.

## Supplementary Materials

The following supporting information can be downloaded at: https://www.mdpi.com/article/10.3390/children13101419/s1, Figure S1: Predicted probability of late-onset hyponatremia by proportion of donor breast milk (DBM) intake, estimated from the multivariable logistic regression model; Table S1: Growth Characteristics of infants; Table S2: Sensitivity and Subgroup Analyses for the Association between Donor Breast Milk Intake and Late-Onset Hyponatremia.

## Abbreviations

The following abbreviations were used in the manuscript

LOH	Late-onset hyponatremia
BW	Birth weight
GA	Gestational age
NICU	Neonatal Intensive Care Unit
PMA	Post-menstrual age
EBM	Expressed breastmilk
DBM	Donor breastmilk
VLBW	Very low birth weight
BPD	Bronchopulmonary dysplasia
RDS	Respiratory distress syndrome
NEC	Necrotizing enterocolitis
hs-PDA	Hemodynamically significant Patent ductus arteriosus
MCT	Medium chain triglyceride
PRBC	Packed red blood cells
OR	Odds Ratio
CI	Confidence Interval
HC	Head Circumference
