# Supplementary material for: The Association of Late-Onset Hyponatremia with Feeding Type in Preterm Infants: A Retrospective Study
Source: Children (Basel). 2026 Oct 16;13(10):1419. doi: 10.3390/children13101419 (PMC13703255; doi:10.3390/children13101419)
Supplement: Supplementary file 1 [file children-13-01419-s001.zip › children-4588687-supplementary.pdf]

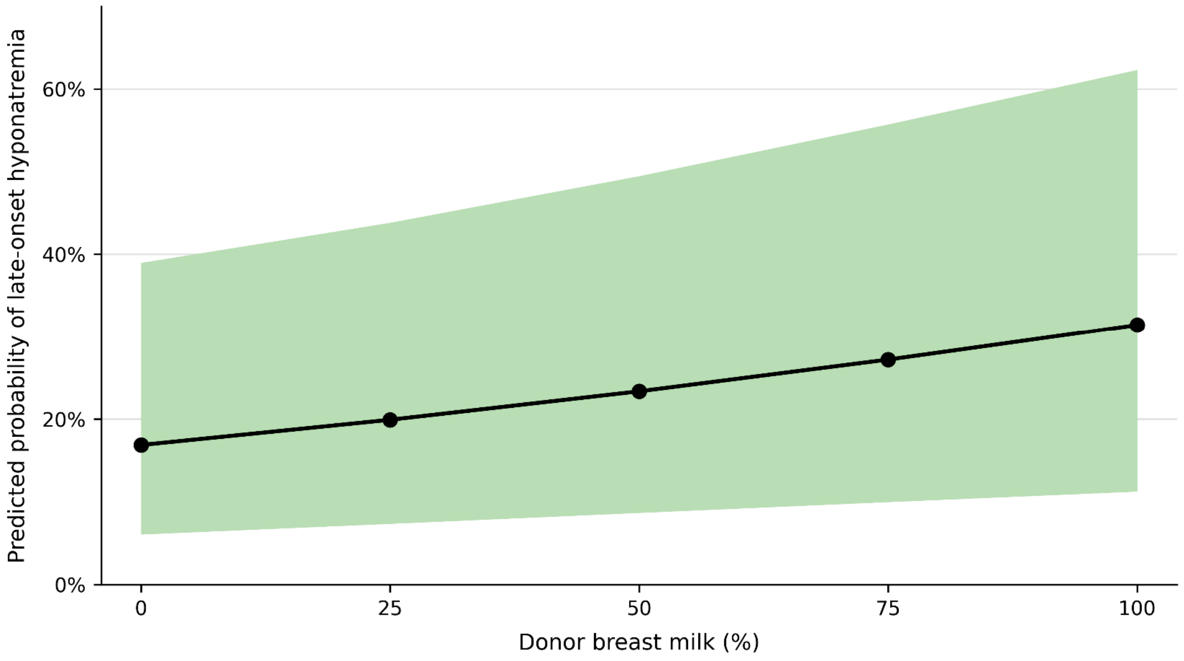

Supplementary Figure S1. Predicted probability of late-onset hyponatremia by proportion of donor breast milk (DBM) intake, estimated from the multivariable logistic regression model. Points show the predicted probability at 0%, 25%, 50%, 75% and 100% DBM; the shaded band shows the 95% confidence interval. Predictions hold formula at 0%, gestational age at the cohort median (28.6 weeks), race at white, and furosemide, chlorothiazide, BPD and PDA as absent.

Table S1. Growth characteristics of infants.

**Variables**	**Total** $N = 373$[1]	**No LOH** $N = 214$[1]	**LOH** $N = 159$[1]	***p*-value**[2]
Weight (birth) Z-score	-0.21 (-0.86, 0.40)	-0.24 (-0.88, 0.34)	-0.18 (-0.81, 0.44)	0.22
Weight (36 weeks PMA) Z-score	-0.99 (-1.65, -0.37)	-0.99 (-1.67, -0.37)	-1.00 (-1.63, -0.39)	0.87
Length (birth) Z-score	-0.28 (-1.08, 0.35)	-0.27 (-1.08, 0.39)	-0.31 (-1.06, 0.31)	0.73
Length (36 weeks PMA) Z-score	-1.17 (-1.85, -0.64)	-1.13 (-1.75, -0.44)	-1.23 (-2.11, -0.83)	0.035
HC (birth) Z-score*	-0.26 (-0.96, 0.49)	-0.30 (-0.97, 0.42)	-0.23 (-0.94, 0.56)	0.29
HC (36 weeks PMA) Z-score	-1.18 (-1.90, -0.49)	-1.00 (-1.71, -0.30)	-1.32 (-1.99, -0.64)	0.003
Weight Z-score change	-0.77 (-1.09, -0.44)	-0.74 (-1.04, -0.43)	-0.82 (-1.23, -0.47)	0.067
Length Z-score change	-0.90 (-1.28, -0.35)	-0.85 (-1.17, -0.27)	-1.00 (-1.48, -0.50)	0.003
HC Z-score change	-0.86 (-1.49, -0.37)	-0.72 (-1.25, -0.24)	-1.20 (-1.78, -0.58)	<0.001

[1]Values are median (IQR). [2]Wilcoxon rank sum test; Pearson's Chi-squared test; Fisher's exact test.
*HC: head circumference.

Table S2. Sensitivity and Subgroup Analyses for the Association between Donor Breast Milk Intake and Late-Onset Hyponatremia.

Model	Analysis	*N*	OR per 1% DBM	95% CI per 1%	OR per 10% DBM	95% CI per 10%	*p*-value
Primary	Full cohort analysis	373	1.0082	1.0013, 1.0153	1.0848	1.0127, 1.1640	0.0217
Sensitivity 1	Excluding infants on diuretics	283	1.0126	1.0046, 1.0208	1.1329	1.0468, 1.2292	0.0022
Sensitivity 2	Excluding infants on prior sodium supplementation	323	1.0053	0.9978, 1.0128	1.0538	0.9786, 1.1353	0.1655
Subgroup 1	Birth GA ≤28 weeks	166	1.0076	0.9982, 1.0179	1.0784	0.9818, 1.1938	0.1267
Subgroup 2	Birth GA >28 weeks	207	1.0106	0.9986, 1.0225	1.1113	0.9866, 1.2491	0.0765

The diuretic analysis excludes 90 unique infants: 29 received furosemide, 74 received chlorothiazide, and 13 received both drugs (103 total drug exposures). All models adjusted for gestational age, race, furosemide, chlorothiazide, BPD, and hs-PDA where applicable. OR = odds ratio; CI = confidence interval.
